# Supplementary material for: Data-driven forecasting of Flu, RSV, and COVID-19 related outcomes in the United States and Canada via Hankel dynamic mode decomposition
Source: PLoS One. 2026 Oct 23;21(10):e0360068. doi: 10.1371/journal.pone.0360068 (PMC13693947; doi:10.1371/journal.pone.0360068)
Supplement: Supplementary file 1 [file pone.0360068.s001.pdf]

Reviewer 1:

Claims regarding comparative performance, particularly the statement that Hankel-DMD is the best-performing model in Canada, should be more carefully qualified. Please explicitly acknowledge the limited number of comparator models and more clearly define the conditions under which Hankel-DMD performs well.

We thank the reviewer for pointing out the need for more qualified claims when comparing our method's performance. We were indeed aware of the fact that the limited number of comparator models was a major contributor of our method ranking more highly for the Canadian forecasting than for the US forecasting, and we had attempted to highlight this in the abstract ("Hankel-DMD is found to be the best performing model for forecasting influenza, RSV, and COVID-19 in Canada, although only three other models submitted enough forecasts against which to compare"). However, we agree with the reviewer that we could be more careful and we have now added several additional statements that we hope better contextualize our results, given the number of available comparator models (Introduction and Discussion).

We have additionally tried to highlight that one of our major contributions is pointing out where and when our method works well, and where and when it works poorly. We believe there are several appealing aspects to using data-driven dynamical systems theory modeling (e.g., lower computational cost, greater interpretability) that motivate its broader use in the field of infectious disease forecasting, but our work shows that more development is necessary before it can be used reliably. Furthermore, our work points to one possible route forward, which is using Dynamic Mode Decomposition with Memory ("DMD with Memory").

Reviewer 2:

Overall, I find that the authors significantly oversell the merits of the method. They praise its performance, but also comment repeatedly that their method offers relatively good forecasts in the early and late stages of an outbreak, but performs poorly in the "middle" (near the peak). Put differently, when the outbreak is clearly growing, the model can predict continued growth, and when the outbreak is clearly declining, the model can predict continued decline. However, it does not capture the actual change point, and leads to very substantial overestimation of e.g. peak sizes. This essentially means that the model captures the "easy" part of the problem (predicting continued growth/decline), but not the challenging bit (predicting slowing growth, the eventual change point, and ensuing reversal of trends).

We thank the reviewer for pointing out the need to be more careful in how we discuss the performance of our method. While we agree that predicting the early and late stages of a given infectious disease season are the "easy" parts of the problem, this does not make the performance of our method trivial. Indeed, as noted by the official FluSight 2024-2025 evaluation release (https://www.cdc.gov/flu-forecasting/evaluation/2024-2025-report.html), there was a significant under-forecasting of US Flu cases in January 2025 by many models. As we show in Fig. 2 of our submission, our method does not have this issue and it outperforms CMU-TimeSeries on this early part of the US Flu season. Furthermore, we find that the two other models that submitted a sufficient number of forecasts to AI4CastingHub did not perform with as high accuracy as our method during the early part of the Canadian Flu season (Fig. 5). Taken together, this demonstrates that – while it may be an "easy" part of the season to model – our method does better on forecasting the early part of the Flu season than some other existing models. Given that FluSight and AI4CastingHub create forecasts by ensembling across individual model forecasts, we believe our method could provide some "counter-balance", which could improve the ensemble performance during the early part of the Flu season. We have now added some additional discussion of this to our submission (Discussion).

All that being said, we recognize that modeling the peak of a given infectious disease is an especially important target of forecasting. Our method's poor performance during the peak highlights the fact that significantly more development is needed to equip Koopman operator-theoretic models with the ability to forecast non-stationary infectious disease trajectories. We have tried to emphasize this failure more in our submission (Introduction and Discussion).

Finally, we note that the need for improving the performance of our method around the peak of a given infectious disease season led us to consider Dynamic Mode Decomposition with Memory (DMD with Memory) and to explore how it could be used for forecasting infectious diseases. While considerably more work needs to be done to fully assess the utility of this method, we note that it can – in certain cases – significantly improve forecasting quality around the peak (e.g., Fig. 4). We therefore view another contribution of our work as highlighting a possible route to improving the performance of Koopman operator-theoretic models on non-stationary data. We have now added more emphasis on this (Abstract).
