# Supplementary material for: Data-driven forecasting of Flu, RSV, and COVID-19 related outcomes in the United States and Canada via Hankel dynamic mode decomposition
Source: PLoS One. 2026 Oct 23;21(10):e0360068. doi: 10.1371/journal.pone.0360068 (PMC13693947; doi:10.1371/journal.pone.0360068)
Supplement: Supplementary file 2 [file pone.0360068.s002.pdf]

General response

We thank the reviewers for their time and their feedback. We are pleased to hear that the work was found to be well motivated and (potentially) useful. We have tried to respond to all of the identified weaknesses of the paper. In particular, we have weakened/qualified some of our claims, added additional emphasis on limitations, and added discussion on how DMD with memory can be integrated in a more automated manner. We respond to each reviewer's point individually, below.

Reviewer 1

1. The current manuscript does not quite provide sufficient guidance on how such periods would be identified prospectively in real time. At present, the proposed remedy appears to rely on retrospective identification of problematic windows and, in places, on the idea that other models in an ensemble may provide counterbalance. I encourage the authors to provide a more explicit discussion of how one might detect, in real time, when the model's predictions are likely to be unreliable, and what practical diagnostics or decision rules could trigger reduced trust in the forecast or a switch to an alternative modeling strategy.

We agree that deciding when to implement DMD with memory is an important point when considering the practical implications of our work. We have now added several sentences in the Discussion of the revised manuscript describing possible ways in which the switching between DMD and DMD with memory can be implemented. This includes both directly using the DMD with memory forecasts when the DMD forecasts are too large (and would prompt a thresholding) and dynamically changing the thresholds for determining a dynamical match, which - when very strict - would be equivalent to using DMD and - when loose - would be equivalent to using DMD with memory on most regions. To further emphasize this important point, we have added language in the Abstract to make it clear that it is the optimal use of DMD with memory that remains to be tested on live forecasting.

Reviewer 2

1. The performance claims should be more carefully qualified. The method appears to perform well during monotonic growth and decline phases, but it performs poorly near epidemic peaks, which are arguably the most important periods for public-health forecasting. In the FluSight analysis, the full-season performance of the vanilla Hankel-DMD model is worse than baseline because of large peak-period errors. This limitation should be presented as a central result, not a secondary caveat.

We agree with the need to be more careful in how we frame the DMD results. We have added language in the Introduction and Discussion of the revised manuscript to make the failure of DMD during the peak of the season a more central result, and not a caveat.

2. The Canadian "best-performing model" claim should be softened. The AI4CastingHub comparison includes only a small number of eligible comparator models. The result is still worth reporting, but the manuscript should state clearly that Hankel-DMD was best among the limited submitted models evaluated, not that it is generally superior to competing forecasting approaches.

The limited number of models that submitted to AI4CastingHub is indeed an important limitation in our assessment of DMD, and we have now made this more clear in the Abstract, Introduction, and Discussion of the revised manuscript.

3. The related-work discussion should cite relevant prior studies. Literature review need to be enriched. For example, Li et al. (2023) proposed deep learning-based COVID-19 forecasting using temporal convolutional networks: DOI: 10.7189/jogh.13.03029. Huang et al. (2021) are most relevant when discussing non-stationarity, interventions, and regional heterogeneity in COVID-19 transmission. Their model not aims on prediction but can certainly be applied for prediction of COVID counts, DOI: 10.1016/j.eclinm.2021.101035. Guo et al. (2022) an example of broader COVID-19 modeling across biological and population scales. DOI: 10.1093/bib/bbab455.

We thank the author for pointing out these relevant works. We have added them to the Introduction of the revised manuscript.

4. The DMD-with-memory analysis needs clearer operational framing. The retrospective results are promising, but it is not clear that the same gains would be achievable in real time, since the approach appears to require knowing when to deploy memory and how to tune parameters. The authors should clarify which parts of this procedure are prospectively implementable and discuss how model switching could be automated.

We agree that deciding when to implement DMD with memory is an important point when considering the practical implications of our work. We have now added several sentences in the Discussion of the revised manuscript describing possible ways in which the switching between DMD and DMD with memory can be implemented. This includes both directly using the DMD with memory forecasts when the DMD forecasts are too large (and would prompt a thresholding) and dynamically changing the thresholds for determining a dynamical match, which when very strict would be equivalent to using DMD and when loose would be equivalent to using DMD with memory on most regions. To further emphasize this important point, we have added language in the Abstract to make it clear that it is the optimal use of DMD with memory that remains to be tested on live forecasting.

5. Thresholding should be treated as an ad hoc correction. Capping forecasts improves performance, but it is not part of the core DMD model and may fail in unusually severe seasons. The authors should either separate thresholded results clearly from the main model evaluation or discuss more principled alternatives, such as spectral regularization or constrained forecasting.

We agree with the reviewer that thresholding is an inelegant and ad hoc correction, and should be avoided when possible. However, we are unsure as to what the reviewer means by separating the thresholded results from the main model - in our manuscript, all retrospective analysis included DMD, DMD with thresholding, and DMD with memory separately (Tables 1, 5-7). In the case of FluSight, we additionally include DMD with memory thresholded (Table 1). For the AI4CastingHub data, we did not include memory with thresholding, as thresholding had little impact (Tables 2-4).

6. Uncertainty quantification needs stronger justification. The Gaussian perturbation strategy for generating quantile forecasts is heuristic. Since probabilistic scoring is central to the evaluation, the authors should provide more calibration evidence or discuss the limitations more explicitly.

We agree that the uncertainty quantification of our submitted forecasts was a heuristic (and indeed, we note this in our manuscript). However, in practice we found this method worked reasonably well. In particular, the 50% and 95% coverage for FluSight was 52% and 86%, respectively (https://www.cdc.gov/flu-forecasting/evaluation/2024-2025-report.html). While we continue to view more principled error quantification as an important direction for future DMD and DMD with memory forecast algorithms, we do not believe the approach we used was a major limiting point of the analyzed season. We have added discussion of this to the Discussion of the revised manuscript.

7. The Canadian data-loading error should be handled more consistently. Two AI4CastingHub submissions were affected by an error. The manuscript should clearly distinguish official submitted results from corrected retrospective analyses, so readers can interpret the tables and figures without ambiguity.

We thank the reviewer for pointing out this confusion. We have now more explicitly stated that the retrospective analysis, which compared DMD and DMD with memory, did not use the two weeks that had data loaded in error. We have additionally added a note of this to Figures 9-11 of the revised manuscript.
