# Supplementary material for: Data-driven forecasting of Flu, RSV, and COVID-19 related outcomes in the United States and Canada via Hankel dynamic mode decomposition
Source: PLoS One. 2026 Oct 23;21(10):e0360068. doi: 10.1371/journal.pone.0360068 (PMC13693947; doi:10.1371/journal.pone.0360068)
Supplement: Peer review history

# Author comment: Author response to Decision Letter 0

**DOI:** 10.1371/journal.pone.0360068.r001

**Transfer Alert**

This paper was transferred from another journal. As a result, its full editorial history (including decision letters, peer reviews and author responses) may not be present.

28 Jan 2026

Attachment Submitted filename: Rebuttal letter.pdf

# Peer review: Decision Letter 0

**DOI:** 10.1371/journal.pone.0360068.r002

**Authors:** Atsushi Asakura

4 May 2026

Dear Dr. Redman,

Thank you for submitting your manuscript to PLOS ONE. After careful consideration, we feel that it has merit but does not fully meet PLOS ONE’s publication criteria as it currently stands. Therefore, we invite you to submit a revised version of the manuscript that addresses the points raised during the review process.

Please submit your revised manuscript by Jun 18 2026 11:59PM. If you will need more time than this to complete your revisions, please reply to this message or contact the journal office at plosone@plos.org. When you're ready to submit your revision, log on to https://www.editorialmanager.com/pone/ and select the 'Submissions Needing Revision' folder to locate your manuscript file.

- A letter that responds to each point raised by the academic editor and reviewer(s). You should upload this letter as a separate file labeled 'Response to Reviewers'.
- A marked-up copy of your manuscript that highlights changes made to the original version. You should upload this as a separate file labeled 'Revised Manuscript with Track Changes'.
- An unmarked version of your revised paper without tracked changes. You should upload this as a separate file labeled 'Manuscript'.

If you would like to make changes to your financial disclosure, please include your updated statement in your cover letter. Guidelines for resubmitting your figure files are available below the reviewer comments at the end of this letter.

If applicable, we recommend that you deposit your laboratory protocols in protocols.io to enhance the reproducibility of your results. Protocols.io assigns your protocol its own identifier (DOI) so that it can be cited independently in the future. For instructions see: https://journals.plos.org/plosone/s/submission-guidelines#loc-laboratory-protocols. Additionally, PLOS ONE offers an option for publishing peer-reviewed Lab Protocol articles, which describe protocols hosted on protocols.io. Read more information on sharing protocols at https://plos.org/protocols?utm_medium=editorial-email&utm_source=authorletters&utm_campaign=protocols.

As the corresponding author, your ORCID iD is verified in the submission system and will appear in the published article. PLOS supports the use of ORCID, and we encourage all coauthors to register for an ORCID iD and use it as well. Please encourage your coauthors to verify their ORCID iD within the submission system before final acceptance, as unverified ORCID iDs will not appear in the published article. *Only*  the individual author can complete the verification step; PLOS staff *cannot*  verify ORCID iDs on behalf of authors.

We look forward to receiving your revised manuscript.

Kind regards,

Atsushi Asakura, Ph.D

Academic Editor

PLOS One

Journal Requirements:

When submitting your revision, we need you to address these additional requirements.

1. Please ensure that your manuscript meets PLOS ONE's style requirements, including those for file naming. The PLOS ONE style templates can be found at

https://journals.plos.org/plosone/s/file?id=wjVg/PLOSOne_formatting_sample_main_body.pdf and

https://journals.plos.org/plosone/s/file?id=ba62/PLOSOne_formatting_sample_title_authors_affiliations.pdf

2. Please note that PLOS One has specific guidelines on code sharing for submissions in which author-generated code underpins the findings in the manuscript. In these cases, we expect all author-generated code to be made available without restrictions upon publication of the work. Please review our guidelines at https://journals.plos.org/plosone/s/materials-and-software-sharing#loc-sharing-code and ensure that your code is shared in a way that follows best practice and facilitates reproducibility and reuse.

3. Thank you for stating the following financial disclosure:

“This study was made possible by the Insight Net cooperative agreement CDC-RFA-FT-23-0069 from the CDC’s Center for Forecasting and Outbreak Analytics. Its contents are solely the responsibility of the authors and do not necessarily represent the

official views of the Centers for Disease Control and Prevention.”

Please state what role the funders took in the study.  If the funders had no role, please state: "The funders had no role in study design, data collection and analysis, decision to publish, or preparation of the manuscript."

If this statement is not correct you must amend it as needed.

Please include this amended Role of Funder statement in your cover letter; we will change the online submission form on your behalf.

4. If the reviewer comments include a recommendation to cite specific previously published works, please review and evaluate these publications to determine whether they are relevant and should be cited. There is no requirement to cite these works unless the editor has indicated otherwise.

[Note: HTML markup is below. Please do not edit.]

Reviewers' comments:

Reviewer's Responses to Questions

**Comments to the Author**

1. Is the manuscript technically sound, and do the data support the conclusions?

Reviewer #1: Yes

Reviewer #2: Yes

**********

2. Has the statistical analysis been performed appropriately and rigorously?

Reviewer #1: Yes

Reviewer #2: Yes

**********

3. Have the authors made all data underlying the findings in their manuscript fully available?

The PLOS Data policy

Reviewer #1: Yes

Reviewer #2: Yes

**********

4. Is the manuscript presented in an intelligible fashion and written in standard English?

Reviewer #1: Yes

Reviewer #2: Yes

**********

Reviewer #1: Overall, this is a thoughtful, interesting, and practically relevant manuscript. I found the paper to be clearly written, technically serious, and valuable in part because it evaluates the method in a live forecasting setting rather than relying solely on retrospective benchmarks. The manuscript is also commendably transparent about where Hankel-DMD performs well and where it struggles, especially around peaks and regime transitions. I also appreciate the authors’ efforts to better qualify claims about comparative performance in Canada, given the limited number of comparator models.

I am favorable toward publication, and I think the manuscript would be strengthened by a few minor revisions. Namely, the manuscript convincingly shows that Hankel-DMD is least reliable around peaks, change points, and other transitions between dynamical regimes, and the authors demonstrate that DMD with memory can improve performance in those periods retrospectively. However, the current manuscript does not quite provide sufficient guidance on how such periods would be identified prospectively in real time. At present, the proposed remedy appears to rely on retrospective identification of problematic windows and, in places, on the idea that other models in an ensemble may provide counterbalance. I encourage the authors to provide a more explicit discussion of how one might detect, in real time, when the model’s predictions are likely to be unreliable, and what practical diagnostics or decision rules could trigger reduced trust in the forecast or a switch to an alternative modeling strategy. Even if a complete solution is outside the scope of the present study, the manuscript would be strengthened by offering a clearer perspective on possible approaches to this problem. This point seems especially important because the manuscript itself acknowledges that selectively using DMD with memory was straightforward retrospectively but that doing so in real time “requires more consideration,” and the limitations section similarly notes that identifying when to deploy DMD with memory should be guided by more principled methods.

Overall, I think this is a strong and useful manuscript. Its main contribution is not that the method solves infectious disease forecasting broadly, but that it offers a clear and honest live benchmark of Hankel-DMD, identifies the settings in which it is promising, and highlights an interesting possible path forward via DMD with memory. With these minor revisions, I think the paper is suitable for publication.

Reviewer #2: This manuscript presents a timely prospective evaluation of Hankel dynamic mode decomposition (DMD) for forecasting influenza, RSV, and COVID-19 outcomes in the United States and Canada. I found the work well motivated and potentially useful. A particular strength is that the authors evaluate the method through real-time submissions to FluSight and AI4CastingHub rather than relying only on retrospective benchmarking. The manuscript is also commendably transparent about important failure modes, especially the tendency of Hankel-DMD to overestimate epidemic peaks. Overall, the study could make a useful contribution, but following major concerns need to be well addressed:

1. The performance claims should be more carefully qualified. The method appears to perform well during monotonic growth and decline phases, but it performs poorly near epidemic peaks, which are arguably the most important periods for public-health forecasting. In the FluSight analysis, the full-season performance of the vanilla Hankel-DMD model is worse than baseline because of large peak-period errors. This limitation should be presented as a central result, not a secondary caveat.

2. The Canadian “best-performing model” claim should be softened. The AI4CastingHub comparison includes only a small number of eligible comparator models. The result is still worth reporting, but the manuscript should state clearly that Hankel-DMD was best among the limited submitted models evaluated, not that it is generally superior to competing forecasting approaches.

3. The related-work discussion should cite relevant prior studies. Literature review need to be enriched. For example, Li et al. (2023) proposed deep learning-based COVID-19 forecasting using temporal convolutional networks: DOI: 10.7189/jogh.13.03029. Huang et al. (2021) are most relevant when discussing non-stationarity, interventions, and regional heterogeneity in COVID-19 transmission. Their model not aims on prediction but can certainly be applied for prediction of COVID counts, DOI: 10.1016/j.eclinm.2021.101035. Guo et al. (2022) an example of broader COVID-19 modeling across biological and population scales. DOI: 10.1093/bib/bbab455.

4. The DMD-with-memory analysis needs clearer operational framing. The retrospective results are promising, but it is not clear that the same gains would be achievable in real time, since the approach appears to require knowing when to deploy memory and how to tune parameters. The authors should clarify which parts of this procedure are prospectively implementable and discuss how model switching could be automated.

5. Thresholding should be treated as an ad hoc correction. Capping forecasts improves performance, but it is not part of the core DMD model and may fail in unusually severe seasons. The authors should either separate thresholded results clearly from the main model evaluation or discuss more principled alternatives, such as spectral regularization or constrained forecasting.

6. Uncertainty quantification needs stronger justification. The Gaussian perturbation strategy for generating quantile forecasts is heuristic. Since probabilistic scoring is central to the evaluation, the authors should provide more calibration evidence or discuss the limitations more explicitly.

7. The Canadian data-loading error should be handled more consistently. Two AI4CastingHub submissions were affected by an error. The manuscript should clearly distinguish official submitted results from corrected retrospective analyses, so readers can interpret the tables and figures without ambiguity.

**********

what does this mean?). If published, this will include your full peer review and any attached files.

If you choose “no”, your identity will remain anonymous but your review may still be made public.

**Do you want your identity to be public for this peer review?**  For information about this choice, including consent withdrawal, please see our Privacy Policy

Reviewer #1: No

Reviewer #2: No

**********

[NOTE: If reviewer comments were submitted as an attachment file, they will be attached to this email and accessible via the submission site. Please log into your account, locate the manuscript record, and check for the action link "View Attachments". If this link does not appear, there are no attachment files.]

To ensure your figures meet our technical requirements, please review our figure guidelines: https://journals.plos.org/plosone/s/figures

You may also use PLOS’s free figure tool, NAAS, to help you prepare publication quality figures: https://journals.plos.org/plosone/s/figures#loc-tools-for-figure-preparation.

NAAS will assess whether your figures meet our technical requirements by comparing each figure against our figure specifications.

# Author comment: Author response to Decision Letter 1

**DOI:** 10.1371/journal.pone.0360068.r003

20 Jul 2026

See attached document with response to reviewers

Attachment Submitted filename: DMD_response_to_reviewers.pdf

# Peer review: Decision Letter 1

**DOI:** 10.1371/journal.pone.0360068.r004

**Authors:** Hans Kestler

22 Sep 2026

Data-driven forecasting of Flu, RSV, and COVID-19 related outcomes in the United States and Canada via Hankel dynamic mode decomposition

PONE-D-26-04818R1

Dear Dr. Redman,

We’re pleased to inform you that your manuscript has been judged scientifically suitable for publication and will be formally accepted for publication once it meets all outstanding technical requirements.

Within one week, you’ll receive an e-mail detailing the required amendments. When these have been addressed, you’ll receive a formal acceptance letter and your manuscript will be scheduled for publication.

An invoice will be generated when your article is formally accepted. Please note, if your institution has a publishing partnership with PLOS and your article meets the relevant criteria, all or part of your publication costs will be covered. Please make sure your user information is up-to-date by logging into Editorial Manager at Editorial Manager® and clicking the ‘Update My Information' link at the top of the page. For questions related to billing, please contact billing support.

If your institution or institutions have a press office, please notify them about your upcoming paper to help maximize its impact. If they’ll be preparing press materials, please inform our press team as soon as possible -- no later than 48 hours after receiving the formal acceptance. Your manuscript will remain under strict press embargo until 2 pm Eastern Time on the date of publication. For more information, please contact onepress@plos.org.

Kind regards,

Hans A. Kestler

Academic Editor

PLOS One

Additional Editor Comments (optional):

Reviewers' comments:

Reviewer's Responses to Questions

**Comments to the Author**

Reviewer #1: All comments have been addressed

Reviewer #2: All comments have been addressed

**********

2. Is the manuscript technically sound, and do the data support the conclusions?

Reviewer #1: Yes

Reviewer #2: Yes

**********

3. Has the statistical analysis been performed appropriately and rigorously?

Reviewer #1: Yes

Reviewer #2: Yes

**********

4. Have the authors made all data underlying the findings in their manuscript fully available?

The PLOS Data policy

Reviewer #1: Yes

Reviewer #2: Yes

**********

5. Is the manuscript presented in an intelligible fashion and written in standard English?

Reviewer #1: Yes

Reviewer #2: Yes

**********

Reviewer #1: (No Response)

Reviewer #2: (No Response)

**********

what does this mean?). If published, this will include your full peer review and any attached files.

If you choose “no”, your identity will remain anonymous but your review may still be made public.

**Do you want your identity to be public for this peer review?**  For information about this choice, including consent withdrawal, please see our Privacy Policy

Reviewer #1: No

Reviewer #2: No

**********

# Editor report: Acceptance letter

**DOI:** 10.1371/journal.pone.0360068.r005

**Authors:** Hans Kestler

PONE-D-26-04818R1

PLOS One

Dear Dr. Redman,

I'm pleased to inform you that your manuscript has been deemed suitable for publication in PLOS One. Congratulations! Your manuscript is now being handed over to our production team.

At this stage, our production department will prepare your paper for publication. This includes ensuring the following:

* All references, tables, and figures are properly cited

* All relevant supporting information is included in the manuscript submission,

* There are no issues that prevent the paper from being properly typeset

You will receive further instructions from the production team, including instructions on how to review your proof when it is ready. Please keep in mind that we are working through a large volume of accepted articles, so please give us a few days to review your paper and let you know the next and final steps.

Lastly, if your institution or institutions have a press office, please let them know about your upcoming paper now to help maximize its impact. If they'll be preparing press materials, please inform our press team within the next 48 hours. Your manuscript will remain under strict press embargo until 2 pm Eastern Time on the date of publication. For more information, please contact onepress@plos.org.

You will receive an invoice from PLOS for your publication fee after your manuscript has reached the completed accept phase. If you receive an email requesting payment before acceptance or for any other service, this may be a phishing scheme. Learn how to identify phishing emails and protect your accounts at https://explore.plos.org/phishing.

If we can help with anything else, please email us at customercare@plos.org.

Thank you for submitting your work to PLOS One and supporting open access.

Kind regards,

PLOS One Editorial Office Staff

on behalf of

Prof. Hans A. Kestler

Academic Editor

PLOS One
